# [Corrigendum] Cancer-associated fibroblasts from invasive breast cancer have an attenuated capacity to secrete collagens

**DOI:** 10.3892/ijo.2024.5675

**Published:** 2024-07-29

**Authors:** Zhixuan Fu, Peiming Song, Dongbo Li, Chenghao Yi, Huarong Chen, Shuqin Ruan, Zhong Shi, Wenhong Xu, Xianhua Fu, Shu Zheng

Int J Oncol 45: 1479-1488, 2014; DOI: 10.3892/ijo.2014.2562

Subsequently to the publication of the above article, an interested reader drew to the authors' attention that the GAPDH bands shown for the western blots portrayed in Fig. 2 (associated with the α-SMA proteins) on p. 1482 were strikingly similar to the GAPDH bands associated with the CAF64 and NF64 experiments in Fig. 4 on p. 1485.

After re-examining their original data, the authors have realized that the GAPDH protein bands correctly shown in Fig. 4 had inadvertently been included in Fig. 2. The revised version of Fig. 2, showing the GAPDH bands that were correctly associated with the α-SMA proteins, is shown opposite. The authors are grateful to the Editor of *International Journal of Oncology* for allowing them this opportunity to publish a Corrigendum, and all the authors agree to its publication. Note that this error did not grossly affect either the results or the conclusions reported in this study; furthermore, the authors apologize to the readership for any inconvenience caused.

## Figures and Tables

**Figure 2:**
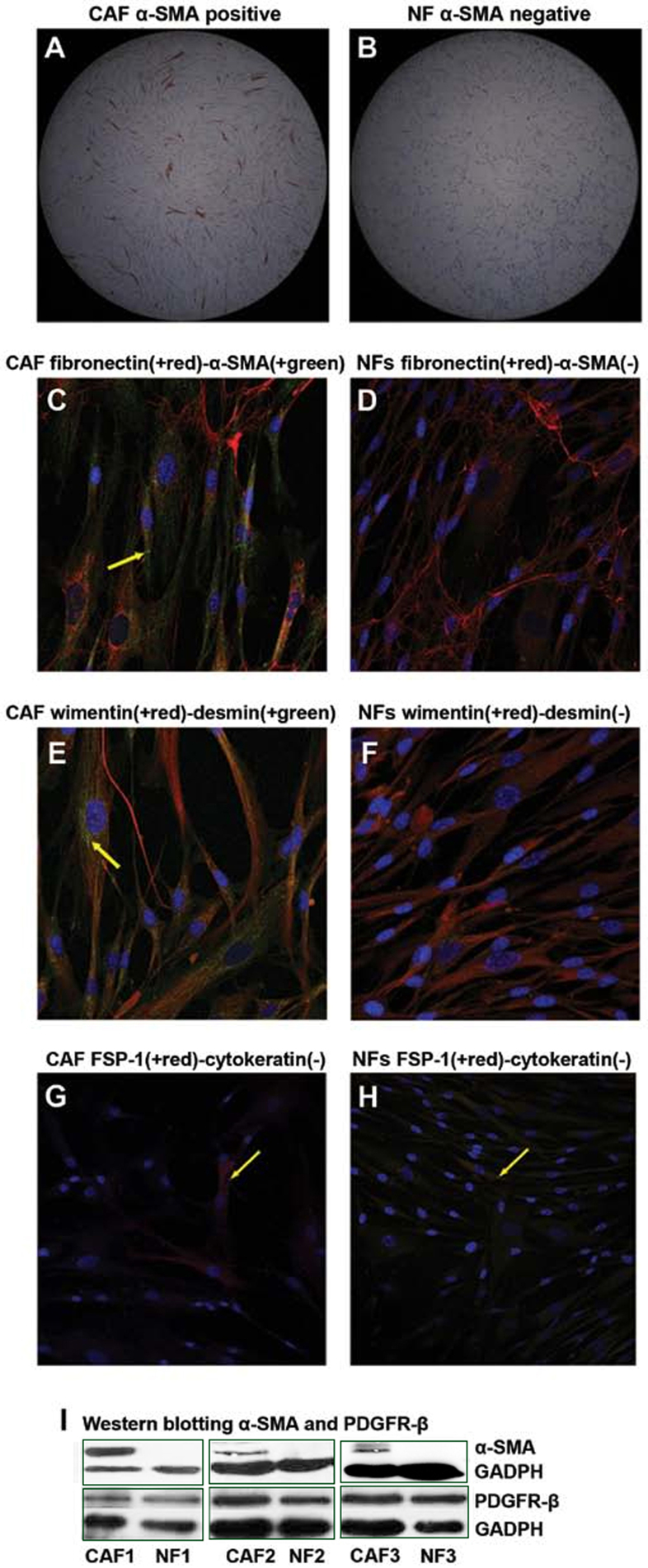
Characterization of CAFs and NFs activation. 1) Immunocytochemistry: The cells were labeled by DAB, fluorescent secondary antibodies (green, red) and DAPI (blue). a) Immunocytochemistry showed CAFs were fractionally α-SMA-positive (A and C), while NF completely lacked α-SMA expression (B and D). b) CAFs and NFs both express vimentin (E and F). In (E), CAFs demonstrated weak expression of desmin, which was absent in NF. c) No expression of cytokeratin (pan) was observed in CAFs or NFs, while the expression of FSP-1 was found only in a small proportion of CAFs and NFs (G and H). (A and B), 50-fold; (C, D, E, F, G and H), 200-fold. 2) Western blot analysis for α-SMA and PDGFR-β assay: CAFs expressed α-SMA, while NF almost completely lacked it. But, there was no expression difference on PDGFR-β between CAFs and NFs (I).

